# Translational signaling and systems biology

**DOI:** 10.1186/s12967-021-03148-y

**Published:** 2021-11-30

**Authors:** Marcello Maggiolini

**Affiliations:** Department of Pharmacy and Health and Nutritional Sciences, University of Calabria, via Bucci, 87036 Rende, Italy

We are delighted to announce the launch of a new section in the Journal of Translational Medicine entitled “Translational signaling and systems biology”.

Cells respond to a broad range of signals sensing transduction mechanisms that feedforward circuits involved in cell life and fate. Great progress has been recently made on how extracellular information are fine tuned into intracellular physiological and pathological outcomes. Regardless of skill to cross (i.e. steroids) or not to cross the cell membrane (i.e. growth factors), extracellular signals are transmitted to intracellular environment through a variety of transduction pathways and sensing cues. Noteworthy, innovative methods and technologies including imaging and nanotechnologies, molecular modelling, new algorithms of artificial intelligence, bioinformatics and systems biology, have better uncovered the actual role of transduction signaling on key cell fitness, from basic to translational research [1–4].

In addition, much attention has been paid on mechanotransduction that refers to the transmission of external mechanical forces to molecular sensors mediating intracellular changes. It is now clear that the complex apparatus harbored by cell membranes not only allows the trafficking of ions and molecules, but also acts as a smart structure in the mechanosensitive processes. For instance, conformational changes and signals originating from the ECM may lead to cell–matrix interactions in peculiar sites named focal adhesions. Then, multi-protein complexes connect extracellular space to the intracellular compartment and the cytoskeleton, turning the transmission of mechanical forces into biological features [5].

Overall, either potential opportunities or problems may be caused by messengers and factors shared by multiple signaling pathways. In this regard, a fine cross-talk among diverse transduction cascades allows a superior regulation of cell activity respect to the action of individual pathways. On the contrary, deleterious cross talk induces certain pitfalls leading to altered biological information. In order to better appreciate this intricate landscape, it appears beneficial to scrutinize the multifaceted signal transduction network by systems biology approaches, which may strongly contribute to clarify how expected or inappropriate biological responses could occur through complex molecular interactions [6]. In this context, the first question is how to assess further causality considering pathways of whole cell by statistical correlation in large data sets. The second issue regards how to unveil novel mechanistic routes crossed by the cell machinery from a systems perspective. Next, modeling and simulation signal transduction systems would provide a unique opportunity to integrate in-vitro and in-vivo experimental data, thus allowing the evaluation of the dynamics that regulate huge molecular platforms. For instance, integrating the mode of action of drugs through dynamical data and a combination of players may be very useful toward the prediction of challenging targeted therapies.

Actually, the field is dynamic and exciting due to the chance to facilitate the translation of new therapeutic breakthroughs targeting dysregulated signaling pathways into clinical settings. Therefore, we are interested in research that investigates and characterizes translational signalling, even taking advantages of systems biology.

The Journal of Translational Medicine provides high standard peer-review process and represents a space for efficiently communicating up-to-date results and scientific discussions. The new section on “Translational signaling and systems biology” will guarantee high quality and competitive publications. The Editorial Board is looking forward to receiving your contributions.
